# Sex-specific differences in fetal and infant growth patterns: a prospective population-based cohort study

**DOI:** 10.1186/s13293-016-0119-1

**Published:** 2016-12-03

**Authors:** Zoe A. Broere-Brown, Esme Baan, Sarah Schalekamp-Timmermans, Bero O. Verburg, Vincent W. V. Jaddoe, Eric A. P. Steegers

**Affiliations:** 1 Department of Obstetrics and Gynecology, Erasmus Medical Center, PO Box 2040, 3000 CA Rotterdam, The Netherlands; 2 Department of Epidemiology, Erasmus Medical Center, Rotterdam, The Netherlands; 3 Department of Pediatrics, Erasmus Medical Center, Rotterdam, The Netherlands

**Keywords:** Fetal sex, Fetal growth, Growth pattern, Biometric indices

## Abstract

**Background:**

The objective of this study was to assess whether sex-specific differences in fetal and infant growth exist.

**Methods:**

This study was embedded in the Generation R Study, a population-based prospective birth cohort. In total, 8556 live singleton births were included. Fetal growth was assessed by ultrasound. During the first trimester, crown-rump-length (CRL) was measured. In the second and third trimester of pregnancy head circumference (HC), abdominal circumference (AC) and femur length (FL) were assessed. Information on infant growth during the first 2 years of life was obtained from Community Health Centers and included HC, body weight and length.

**Results:**

In the first trimester, male CRL was larger than female CRL (0.12 SD [95% CI 0.03,0.22]). From the second trimester onwards, HC and AC were larger in males than in females (0.30 SD [95% CI 0.26,0.34] and 0.09 SD [95% CI 0.05,0.014], respectively). However, FL in males was smaller compared to female fetuses (0.21 SD [95% CI 0.17,0.26]). Repeated measurement analyses showed a different prenatal as well as postnatal HC growth pattern between males and females. A different pattern in body weight was observed with a higher body weight in males until the age of 12 months where after females have a higher body weight.

**Conclusions:**

Sex affects both fetal as well as infant growth. Besides body size, also body proportions differ between males and females with different growth patterns. This sexual dimorphism might arise from differences in fetal programming with sex specific health differences as a consequence in later life.

**Electronic supplementary material:**

The online version of this article (doi:10.1186/s13293-016-0119-1) contains supplementary material, which is available to authorized users.

## Background

Embryonic and fetal growth are dependent on many factors including adequate placental development and function. This can be reflected by several placental biomarkers in maternal plasma such as the pro-angiogenic placental growth factor (PlGF) and the anti-angiogenic soluble fms-like tyrosine kinase 1 (s-Flt1) [1, 2]. Previous studies have shown associations between placental biomarkers and fetal growth [3–5]*.*

Recently, fetal sex-specific differences in placental biomarkers were observed with higher first trimester levels of s-Flt1 and PlGF in women carrying a female fetus. This may suggest that placentation processes differ according to fetal sex [6, 7]. This difference in placental function might influence fetal growth and/or fetal programming in a sex-specific manner. Indeed, previous research has shown fetal sex-specific differences in biometrical indices and growth patterns, and fetal sex-specific growth curves were created [8]. However, these growth curves were based on cross-sectional data and serial measurements of the same fetus were not available. Moreover, it is of interest to investigate whether sex-specific differences in fetal growth persist into infancy since the Development and Origins of Health and Disease (DOHaD) theory states that deviations in early growth are associated with adverse health in later life.

With this study, we investigate whether there are fetal sex-specific differences in fetal and infant growth in a large study population. We repeatedly assessed fetal growth during pregnancy by measuring crown-rump-length (CRL) in the first trimester, and several biometrical indices (head circumference (HC), abdominal circumference (AC) and femur length (FL)) in the second and third trimester of pregnancy. After pregnancy until the age of 2 years, growth was assessed at several time points by assessing HC and body weight and length. In addition, we explore the effect of the presence or absence of the placental syndrome on these differences.

## Methods

### Study design

**Fig. 1:**
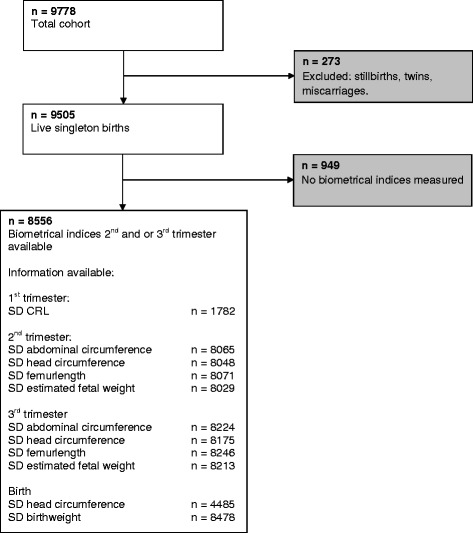
Flowchart

This study was embedded in the Generation R Study, a population-based prospective cohort study from early pregnancy onwards in Rotterdam, The Netherlands [9]. The study is designed to identify early environmental causes of normal and abnormal growth, development and health from fetal life until young adulthood. Eligible mothers were those who were resident in the study area at their delivery date between April 2002 and January 2006. We aimed to enroll mothers in early pregnancy (before 18 weeks of gestation). In total, 9778 mothers were included. For the present study, women with a live singleton birth with at least one prenatally assessed biometric measurement were eligible (Fig. 1). The study has been approved by the Medical Ethics Committee of the Erasmus Medical Center, Rotterdam, The Netherlands. Written informed consent was obtained from all participants.

### Pregnancy dating

Precise initial dating by early ultrasonography is vital to ensure accurate pregnancy dating, especially when assessing fetal growth. Dating of pregnancy was performed using the first ultrasound measurement of either the CRL (if the gestational age was below 12 weeks and 5 days and CRL measurement <65 mm), or the biparietal diameter (BPD) (from a gestational age from 12 weeks and 5 days onwards with a BPD >23 mm). Establishing gestational age with fetal ultrasound examinations is the most accurate method for pregnancy dating [10–12].

### Fetal growth

#### First trimester

Since pregnancy dating was based on CRL, only CRL measurements of women with a known and reliable first day of the last menstrual period (LMP) with a regular cycle (lasting 28 days +/- 4 days) were included in the analyses (*n* = 1782). For the purpose of analyses on CRL measurement, pregnancy dating in this subgroup of women was not based on CRL but on the LMP.

#### Second and third trimester

Fetal ultrasound examinations were performed in the second (median 20.5 weeks of gestation, 90% range 18.9–22.9) and third trimester (median 30.3 weeks of gestation, 90% range 28.7–32.4). Fetal biometry (HC, AC, and FL) was performed trans abdominally during each ultrasound examination. Standardized ultrasound planes for HC, AC, and FL are described elsewhere [13–15]. Estimated fetal weight (EFW) was calculated using the formula of Hadlock with parameters AC, HC, and FL (in cm): EFW = 10**(1.326 −0.00326*AC*FL + 0.0107*HC + 0.0438*AC + 0.158*FL [16].

Gestational age-adjusted standard deviation scores (SDS) were calculated for all fetal growth measurements, including CRL measurements. These gestational age-adjusted standard deviation scores were based on reference growth curves from the whole study population and represent the equivalent of Z-scores [17]. Ultrasound examinations were performed using an Aloka® model SSD-1700 (Tokyo, Japan) or the ATL-Philips® Model HDI 5000 (Seattle, WA, USA).

### Delivery and birth complications

Information on gestational age at birth, offspring sex, and pregnancy complications (pre-eclampsia [PE], a neonate born small for gestational age [SGA] and/or spontaneous preterm birth [sPTB]) was obtained from medical records. sPTB was defined as a spontaneous onset of birth <37 weeks of gestation. SGA was defined as a gestational age and fetal sex-adjusted birthweight below the 10th percentile [17]. Pre-eclampsia was defined as the development of SBP ≥ 140 mmHg and/or DBP ≥ 90 mmHg after 20 weeks of gestation plus the presence of proteinuria (≥0.3 g in a 24-hour urine specimen or ≥2 + [1 g/L] on a voided specimen, or ≥1 + [0.3 g/L] on a catheterized specimen) in previously normotensive women [18].

### Infant growth

Well-trained staff in the Community Health Centers obtained postnatal growth characteristics according to standard schedules and procedures at the median ages of 1.1 (90% range 0.8–1.6), 2.2 (90% range 2.0–2.9), 3.3 (90% range 3.0–3.9), 4.4 (90% range 4.0–4.9), 6.2 (90% range 5.4–7.6), 11.1 (90% range 10.2–12.3), 14.3 (90% range 13.7–15.7), 18.3 (90% range 17.5–20.8) and 24.8 (90% range 23.6–27.5) months. Growth characteristics included body weight, length, and HC. SD scores were obtained with the Dutch growth reference charts (Growth Analyzer 3.0, Dutch Growth Research Foundation, Rotterdam, The Netherlands).

### Statistical analysis

Firstly, we performed student *t* tests and Chi-square tests to test sex-specific differences in fetal growth characteristics. Linear regression analyses were then performed to relate fetal biometric indices to sex. To further explore growth patterns between female and male fetuses and infants, repeated measurement regression models were performed using the mixed model procedure with fetal and infant growth as a repeated outcome measure. These models take the correlation between repeated measurements of the same subject into account. Regarding the repeated measurement analyses that we used to assess fetal growth patterns, we used SDS according to the Niklasson growth standards. This standard adjusts for fetal sex. In addition, we stratified for fetal sex in our analyses, which creates the potential risk of overadjusting. The growth standard of Usher and McLean is to our knowledge the only standard available not adjusting for fetal sex [19]. Repeated measurement analyses on weight using this standard instead of the Niklasson standard are shown in Additional file 1: Figure S1. Finally, to investigate differences in fetal growth in pregnancies with a suboptimal intrauterine environment, we created the composite outcome scores “complicated pregnancy” and “uncomplicated pregnancy”. Pregnancies complicated by either PE and/or sPTB and/or SGA were classified as being complicated. Uncomplicated pregnancies were defined by the absence of all these complications. All abovementioned linear regression and repeated measurement analyses were also performed within strata of these composite scores. Since fetal sex does not have any true confounding factors (e.g., smoking, folic acid intake, maternal ethnicity), primary analyses were not adjusted for any covariates. However, since including these covariates into the analyses may be informative, we included them in additional analyses shown in Additional file 2: Table S1. By using SD scores of all outcomes, we automatically adjusted for gestational age at the time of measurement.

Lastly, effect modification was tested on a multiplicative scale with maternal smoking and ethnicity. If the interaction term was statistically significant, regression or repeated measurement analyses were performed in strata of that specific determinant.

Statistical analyses were performed using either the Statistical Package of Social Sciences version 21.0 for Windows (SPSS Inc., Chicago, IL, USA) or the Statistical Analysis System version 9.3 (SAS, Institute Inc., Gary NC, USA).

## Results

### Study population

Baseline characteristics of all participants are presented in Table 1. There were no differences between women pregnant with a male or female fetus concerning maternal age, height, weight, BMI, ethnicity, educational level, folic acid use, or parity. Women with a male fetus smoked more often (*p* < 0.01). Also gestational age at ultrasound and gestational age at birth differed between male and female fetuses.

**Table 1:** Baseline characteristics stratified by fetal sex

	Total	Females	Males	*p* value
	*n* = 8556	*n* = 4230	*n* = 4326	
Maternal age	29.6 (5.3)	29.6 (5.3)	29.7 (5.3)	0.49
Anthropometrics				
Height (cm)	167.5 (7.4)	167.4 (7.5)	167.6 (7.3)	0.25
Weight (kg)	66.3 (12.9)	66.4 (12.9)	66.1 (12.8)	0.32
BMI (kg/m^2^)	23.9 (19.4–33.8)	24.0 (19.4–33.9)	23.8 (19.3–33.7)	0.12
Ethnicity				0.44
Western	4664 (57.5%)	2316 (57.9%)	2348 (57.1%)	
Non-Western	3447 (42.5%)	1682 (42.1%)	1765 (42.9%)	
Educational level				0.74
Low	907 (11.6%)	462 (12.0%)	445 (11.3%)	
Middle	3627 (46.4%)	1782 (46.2%)	1845 (46.7%)	
High	3275 (41.9%)	1611 (41.8%)	1664 (42.1%)	
Smoking habits				0.004
No	5452 (72.8%)	2740 (73.9%)	2712 (71.6%)	
Yes-stopped	642 (8.6%)	330 (8.9%)	312 (8.2%)	
Yes-continued	1399 (18.7%)	636 (17.2%)	763 (20.1%)	
Folic acid use-yes (%)				0.11
No	1863 (29.3%)	899 (28.5%)	964 (30.2%)	
Before 10 weeks	1978 (31.1%)	971 (30.7%)	1007 (31.5%)	
Preconception start	2512 (39.5%)	1289 (40.8%)	1223 (38.3%)	
Nulliparous (%)	4718 (55.8%)	2353 (56.4%)	2365 (55.2%)	0.89
Gestational age at sonography (wks)				
First trimester	12.5 (11.1–14.5)	12.5 (11.1–14.4)	12.5 (11.1–14.6)	0.04
Second trimester	20.5 (18.9–22.9)	20.4 (18.8–22.8)	20.6 (18.9–23.0)	<0.001
Third trimester	30.3 (28.7–32.4)	30.3 (28.6–32.4)	30.4 (28.9–32.4)	<0.001
Gestational age at birth (wks)	40.1 (36.9–42.0)	40.1 (36.9–42.0)	40.1 (36.7–42.1)	0.001

Data are represented as *n* (%) or as the mean (SD) or as the median with the 90% range

Differences in baseline characteristics were tested using student *t* test, Mann-Whitney U test, and Chi-square test

### Effect of sex on fetal growth

Already in the first trimester, fetal growth differed between the two sexes. Male fetuses had a larger CRL as compared to female fetuses with a difference of 0.12 SD [95% CI 0.03,0.22, *p* < 0.001]. In the second trimester of pregnancy, male fetuses had a lower EFW of 0.05 SD [95% CI 0.00,0.09, *p* = 0.03] calculated with the Hadlock formula. In the third trimester, no differences concerning EFW were observed. At birth, male neonates were on average of 188 g heavier than female neonates [95% CI 182,193, *p* < 0.001].

**Fig. 2:**
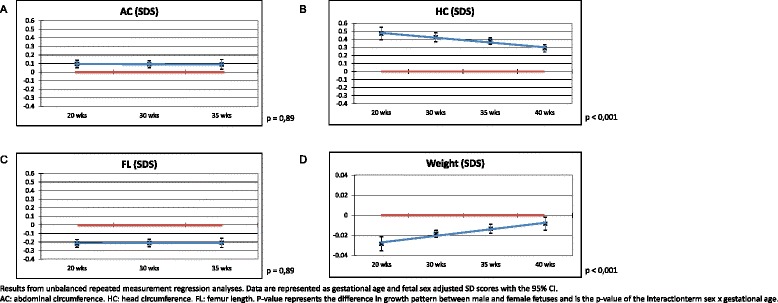
**a**–**d** Associations between sex and fetal growth–repeated measurement analyses adjusted for maternal smoking

In Table 2, the results of the linear regression analyses on biometrical indices are depicted. Male sex was associated with a larger AC and HC, but a smaller FL in both the second and the third trimester of pregnancy (all *p* < 0.001). Results of the repeated measurements in SDS are shown in Fig. 2a–d. Although male fetuses have a larger AC and a smaller FL compared with female fetuses, the growth pattern of AC and FL did not differ between male and female fetuses ( both *p* = 0.89). Additionally, male HC is larger than female HC. This difference changes during pregnancy as the difference between male and female HC decreases as pregnancy precedes. This indicates that the growth pattern of HC differs with fetal sex in which male fetuses have a slower growth rate of HC than female fetuses (*p* < 0.001).

**Table 2:** Linear regression analyses on fetal growth

	AC (SDS)	HC (SDS)	FL (SDS)
	*β*	*β*	*β*
Total			
2nd trimester			
Females	Reference	Reference	Reference
Males	0.09 [0.05, 0.14]***	0.30 [0.26, 0.34]***	−0.21 [−0.26, −0.17]***
3rd trimester			
Females	Reference	Reference	Reference
Males	0.09[0.05, 0.13]***	0.38 [0.34, 0.42]***	−0.21 [−0.26, −0.17]***
Uncomplicated			
2nd trimester			
Females	Reference	Reference	Reference
Males	0.10 [0.05, 0.15]***	0.29 [0.25, 0.34]***	−0.21 [−0.25, −0.16]***
3rd trimester			
Females	Reference	Reference	Reference
Males	0.12 [0.07, 0.16]***	0.38 [0.34, 0.43]***	−0.20 [−0.25, −0.15]***
Complicated			
2nd trimester			
Females	Reference	Reference	Reference
Males	0.09 [−0.03, 0.21]	0.38 [0.26, 0.50]***	−0.26 [−0.38, −0.13]***
3rd trimester			
Females	Reference	Reference	Reference
Males	0.04 [−0.08, 0.17]	0.42 [0.30, 0.54]***	−0.27 [−0.40, −0.15]***

Values are regression coefficients (95% CI) and represent data in SDS

Abbreviations: *AC* abdominal circumference, *HC* head circumference, *FL* femur length

***p < 0.001

Regarding analyses assessing the effects of a suboptimal intrauterine environment, i.e., complicated versus uncomplicated pregnancies, similar trends were observed with larger AC and HC and smaller FL in male fetuses compared to female fetuses (all *p* < 0.001). The only exception is that the AC in the complicated group did not differ between male and female fetuses in both the second and third trimester of pregnancy (respectively *p* = 0.11 and *p* = 0.46).

### Effect of fetal sex on infant growth

**Fig. 3:**
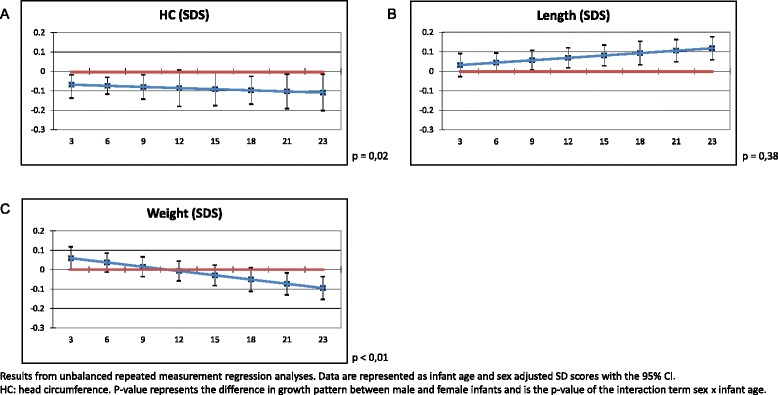
**a**–**c** Associations between sex and infant growth–repeated measurement analyses

The results of the repeated measurements in SDS are shown in Fig. 3a–c. Males have a smaller HC from 3 months onwards. The difference in HC increases with advancing age. The growth pattern of HC was significantly different between the two sexes (*p* = 0.02). Males have a larger body length compared with females. This was statistically significant from 9 months onwards. Although it seems that the difference between males and females increases with advancing age, the pattern in body length between the two sexes was not statistically significant (*p* = 0.38). For the pattern of body weight, a crossover was observed. At the age of 3 months and from 21 months onwards, the difference in body weight was statistically different between males and females. Due to the crossover, body weight patterns were statistically different for females compared with males (*p* < 0.01).

We did not find an interaction with maternal smoking or ethnicity on both fetal and infant growth characteristics.

## Discussion

### Main findings

In this study, we demonstrate fetal sex-specific differences in fetal growth. These differences are already present from the first trimester of pregnancy onwards and track throughout pregnancy. Male and female fetuses do not only differ in weight but also differ in biometric indices with a different body proportion as a consequence. Moreover, male fetuses follow a different growth pattern than female fetuses with a slower growth rate of the HC. The presence of PE, SGA, or PTB does not affect this. During infancy, the difference in HC growth patterns persists and a difference in weight patterns arises.

### Interpretation

The Development and Origins of Health and Disease (DOHaD) theory states that in the case of adverse fetal exposure, the unborn fetus can modify its own development such that it will be prepared for survival in an environment in which resources are likely to be short. These early life adaptations help in survival by selecting an appropriate trajectory of growth in response to the environment. Although these adaptations may be beneficial for short-term survival, they may have adverse consequences at birth or in later life [20, 21]. The sex-specific differences in body proportion and fetal growth shown in this study might therefore be one of the bases for sex differences shown in chronic diseases in later life. Especially, since this study shows that sexual dimorphism in growth persist after birth until the age of two years. Little research has been done on differences in growth patterns between males and females. Some studies performed on a later age acknowledge differences in body proportions [22]. Moreover, peak velocity in growth differs between males and females since the timing of the beginning of puberty and therefore growth spurt is different [23, 24]. This is one of the first studies showing that differences in growth patterns between males and females begin at a very early age.

Fetal sex-specific differences in fetal growth and growth patterns may be explained by differences in placentation as previously suggested by our group [6]. Maternal serum levels of s-Flt1, PLGF, and PAI-2 were shown to be higher in the presence of a female fetus. However, Bouwland-Both et al. from our group showed positive associations between maternal s-Flt1, PLGF, plasminogen activator inhibitor 2 (PAI-2) in early pregnancy and CRL [3]. According to these results, one would expect that female CRL is increased compared with male CRL. However, this study showed that male embryos had a larger CRL as compared with females. Considering the effect of placental biomarkers on embryonic growth and the sex-specific differences in these biomarkers, this conflicting result might be explained by effect modification. Hence, the effect of placental biomarkers on CRL is dependent on fetal sex. For this reason, we performed additional interaction analyses showing that in a male embryo, PAI-2 has a larger effect on CRL SDS than in a female embryo (data not shown). Furthermore, we added our data on placental biomarkers (the first and second trimester s-Flt1 and PLGF) into the statistical models to test possible mediating effects of these biomarkers as a proxy for placentation (Additional file 2: Table S1). Although results remained significant, several effect estimates changed with more than 10%. This implies an intermediate role for placental biomarkers. Moreover, extra-placental sources of these biomarkers exist. Previous studies show that s-Flt1 is also produced by maternal endothelial cells [25]. These extra-placental sources could potentially contribute to the differences in early embryonic growth since these biomarkers are associated with CRL.

Some studies state that fetal growth is the result of the availability of nutrients and therefore is mainly determined by placental function [26]. However, until the 11th week of pregnancy, cytotrophoblast plugs obliterate the tips of the utero-placental arteries preventing blood flow with the consequence that fetal growth is not dependent on hemotrophic nutrition during the first trimester. Hence, it is plausible that in early pregnancy, not only placentation determines fetal growth but also other underlying factors such as intrinsic factors of the fetus (i.e., sex). We observed no differences between male and female fetal growth patterns in complicated and uncomplicated pregnancies. This may indicate that physiological placental regulatory mechanisms may be overruled by the pathophysiological sequelae in pregnancies complicated by PE, SGA, and PTB [6, 27].

Concerning biometrical indices, little research has been performed focusing on fetal sex. One study reported on sex-specific antenatal growth charts [28]. However, these growth charts were based on a population of 4234 women with just one antenatal measurement. Similar with our present study, they showed larger HC and AC in male fetuses. In contrast with our results, they showed that the difference between male and female HC increased with proceeding gestation. They did not show an effect of fetal sex on FL. Another study also assessed fetal sex-specific differences in biometrical indices [29]. They too found a larger HC in the case of a male fetus. This study had a smaller sample size of 427 measurements, and analyses were performed cross-sectional.

In our study, we observed a discrepancy in EFW and birthweight. Birthweight was higher in males, while EFW in the second and third trimester of pregnancy was higher in females. This inconsistency can either be explained by the applied formula estimating EFW or by the method used to determine gestational age of pregnancy. The Hadlock formula uses HC, AC, and FL to calculate the EFW. In our study, male and female fetuses differed in body composition with different indices for male and female fetuses. The Hadlock formula could therefore be improved by adjusting for fetal sex as previously suggested by others [8, 30–33]. Melamed et al. constructed a fetal sex-adjusted Hadlock formula which indeed showed a decrease in the systematic error [34]. Secondly, to determine gestational age of pregnancy, we used either the first trimester CRL or BPD measurement. This study however shows that CRL of a male embryo is larger than that of a female embryo. A possible consequence is that male embryos were dated as being older which might explain why females have a higher EFW during the second and third trimester. Lastly, we have to consider the possibility that the growth rate of female fetuses is relatively high in the second trimester while the growth rate of males is higher in the third trimester. This is confirmed by de Jong et al. who showed that the daily growth rate in the third trimester prior to birth was significantly higher for male fetuses [35]. This would indeed result in a higher EFW in the second trimester but a lower birthweight for female fetuses as seen in this study.

## Conclusions

In conclusion, we can state that there are differences in fetal and infant growth between males and females. These findings help us in the understanding of the mechanism of growth which is not only important for birth outcome but also predisposes for possible adverse adult health. In clinical practice as well as future research concerning placentation and fetal development and growth, sex has to be taken into account.

## Additional files

Additional file 1: Figure S1. Associations between fetal sex and weight– repeated measurements analyses. (PDF 86 kb)
Additional file 2: Table S1. Adjusted linear regression analyses on fetal growth. (XLSX 12 kb)
