# Supplementary material for: Sex-specific differences in fetal and infant growth patterns: a prospective population-based cohort study
Source: Biol Sex Differ. 2016 Dec 3;7:65. doi: 10.1186/s13293-016-0119-1 (PMC5135770; doi:10.1186/s13293-016-0119-1)
Supplement: Additional file 1: Figure S1. — Associations between fetal sex and weight– repeated measurements analyses. (PDF 86 kb) [file 13293_2016_119_MOESM1_ESM.pdf]

Supplemental Figure 1 **Associations between fetal sex and weight– repeated measurements analyses**

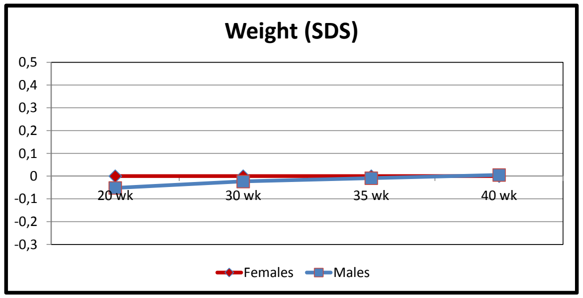

P = 0,10

Results from unbalanced repeated measurement regression analyses. Data are represented as SD scores.
For this analyses the Usher and McLean standard was used.
